# Myogenic progenitors contribute to open but not closed fracture repair

**DOI:** 10.1186/1471-2474-12-288

**Published:** 2011-12-22

**Authors:** Renjing Liu, Oliver Birke, Alyson Morse, Lauren Peacock, Kathy Mikulec, David G Little, Aaron Schindeler

**Affiliations:** 1 Orthopaedic Research & Biotechnology Unit, The Children's Hospital at Westmead, Sydney, Australia; 2 Discipline of Paediatrics and Child Health, Faculty of Medicine, University of Sydney, Sydney, Australia

## Abstract

**Background:**

Bone repair is dependent on the presence of osteocompetent progenitors that are able to differentiate and generate new bone. Muscle is found in close association with orthopaedic injury, however its capacity to make a cellular contribution to bone repair remains ambiguous. We hypothesized that myogenic cells of the MyoD-lineage are able to contribute to bone repair.

**Methods:**

We employed a *MyoD*-Cre^+^:Z/AP^+ ^conditional reporter mouse in which all cells of the MyoD-lineage are permanently labeled with a *human alkaline phosphatase (hAP) *reporter. We tracked the contribution of MyoD-lineage cells in mouse models of tibial bone healing.

**Results:**

In the absence of musculoskeletal trauma, MyoD-expressing cells are limited to skeletal muscle and the presence of reporter-positive cells in non-muscle tissues is negligible. In a closed tibial fracture model, there was no significant contribution of hAP^+ ^cells to the healing callus. In contrast, open tibial fractures featuring periosteal stripping and muscle fenestration had up to 50% of hAP^+ ^cells detected in the open fracture callus. At early stages of repair, many hAP^+ ^cells exhibited a chondrocyte morphology, with lesser numbers of osteoblast-like hAP^+ ^cells present at the later stages. Serial sections stained for hAP and type II and type I collagen showed that MyoD-lineage cells were surrounded by cartilaginous or bony matrix, suggestive of a functional role in the repair process. To exclude the prospect that osteoprogenitors spontaneously express MyoD during bone repair, we created a metaphyseal drill hole defect in the tibia. No hAP^+ ^staining was observed in this model suggesting that the expression of MyoD is not a normal event for endogenous osteoprogenitors.

**Conclusions:**

These data document for the first time that muscle cells can play a significant secondary role in bone repair and this knowledge may lead to important translational applications in orthopaedic surgery.

Please see related article: http://www.biomedcentral.com/1741-7015/9/136

## Background

The conventional cellular understanding of the bone repair process places progenitors originating from the periosteum and the bone marrow compartment in a pivotal role. The periosteum, a cellular layer surrounding bones, has a well defined capacity for bone repair [1]. Mesenchymal progenitors from the marrow are also highly plastic and are involved in bone homeostasis [2]. However, there are many orthopaedic circumstances where damage to the periosteum, debridement to prevent infection, and internal fixation limit the access by these primary osteoprogenitors. High-energy traumatic fractures and open fractures have a much higher relative risk of non-union [3], yet many can still go on to unite. Although one possibility is that the periosteal and/or marrow tissues are able to recover sufficiently to facilitate repair, we have hypothesized that secondary progenitor cell types are likely to compensate.

We have recently reviewed a range of progenitor cell types that may have the potential to contribute to bone repair [4]. These include cells from the adjacent soft tissues (myogenic progenitors, vascular endothelial cells, and pericytes) as well as circulating progenitors. Myogenic cells represent a strong candidate for contributing to local bone healing. In cell culture, myogenic cells are highly responsive to osteogenic growth factors and the osteogenic response of non-myogenic cells can be increased by forced expression of the myogenic factor *MyoD *[5]. Muscle-derived cells cultured *ex vivo *and re-implanted into mice have been shown to be able to contribute to new bone formation [6,7]. When bone repair is observed in the clinical orthopaedic setting, the bone that first forms in response to a fracture is often seen adjacent to the local muscles [8]. Nevertheless, such data is only circumstantial and does not specifically demonstrate that myogenic cells can contribute to bone formation in an *in vivo *setting.

One challenge with examining the contribution of different progenitor populations *in vivo *is tracking their contribution to other tissues once they have ceased expressing markers that define their origin. To overcome this limitation, researchers have started to employ Cre/*loxP *conditional reporter systems where early lineage specification markers can drive a permanent recombination event in all downstream tissues. In this study, we have utilized a *MyoD*-Cre mouse line, where the majority of early myogenic progenitors can be permanently labeled. Prior reports have indicated that the presence of MyoD-lineage cells in non-muscle tissues is a rare event [9], and we have confirmed this experimentally (Additional File 1, Additional File 2, Additional File 3). We determined that this would be a suitable model to examine the contribution of myogenic progenitors to orthopaedic repair.

*MyoD*-Cre and *Tie2*-Cre reporter mice have been previously employed to study the contribution of endogenous myogenic and vascular progenitors in models of heterotopic bone formation [10]. Lounev *et al *reported that the process of rhBMP-2 induced intramuscular bone formation featured a negligible contribution by MyoD-lineage cells, but a major (up to 50%) contribution by Tie2-lineage cells. However, prior work by Lu *et al *indicated a minimal contribution by Tie2-lineage cells to fracture repair [11], suggesting important fundamental differences between heterotopic ossification and fracture healing.

In the present study, we have performed a detailed analysis of the contribution of MyoD-lineage cells to bone repair in the mouse tibia using a number of surgical models. First, we compared closed tibial fractures where the periosteum was largely intact with an open, highly-traumatic fracture model featuring periosteal stripping and local tissue trauma. We hypothesized that the involvement of any secondary repair systems, such as from the myogenic progenitors, would be greatest when the periosteal progenitors were deficient. Next, we examined a drill hole defect model in the proximal tibial metaphysis where all adjacent muscle tissues were stripped. Histological staining for the hAP reporter was used to determine the contribution of the MyoD-lineage cells to the fracture callus or bone defect. Adjacent sections were also co-stained for bone/cartilage matrix markers to confirm the successful transdifferentiation of the once myogenic cells into the osteogenic and chondrogenic lineages.

## Methods

### Mouse lines

The *MyoD*-Cre mouse line expresses the Cre recombinase gene under the control of the *MyoD *promoter [12]. This mouse line was a gift from A/Prof David Goldhamer (University of Connecticut, Storrs, CN, United States). In the absence of Cre recombinase, the Z/AP reporter strain constitutively expresses a *lacZ *transgene; upon exposure to Cre, the transgene recombines to express *human placental alkaline phosphatase *(*hAP*) [13]. This mouse line allows specific distinct between reporter and endogenous expression as hAP is heat-resistant while endogenous alkaline phosphatase is heat-labile. This mouse line was supplied by Prof. Patrick Tam (Children's Medical Research Institute, Westmead, NSW, Australia) with permission from Prof. Andras Nagy (Samuel Lunenfeld Research Institute, Toronto, Ontario, Canada), who originally published the strain. The *MyoD*-Cre mouse line was on a predominantly C57BL6/J background (> 5 generations backcross) and the Z/AP line was of a mixed 129/SvJ:C57BL6/J background. *MyoD*-Cre^+^:Z/AP^+ ^mice were generated by breeding mice heterozygous for the transgenes; *MyoD*-Cre^-^:Z/AP^+ ^littermates were used as staining controls. For developmental experiments, transgenic embryos were produced by timed mating.

Mice were housed at the Children's Hospital at Westmead (CHW) Transgenic Facility and at the Westmead Hospital Department of Animal Care. The health status of mice was routinely monitored by animal house staff and mice were given food and water *ad libitum*. Animal experimentation was approved by the CHW/CMRI Animal Ethics Committee (K248) and the Westmead Hospital Animal Ethics Committee (4102).

### Genotyping

Transgenic mice were genotyped using ear or tail DNA by standard PCR method using Taq polymerase. Tissue samples were digested with Direct PCR lysis buffer (Viagen Biotech, Los Angeles, CA, United States) with added proteinase K (333 μg/ml) at 55°C for 2 hours to overnight. Proteinase K activity was terminated by heat-denaturation at 85°C for 40 min. Samples were run on a 2% TAE agarose gel with ethidium bromide and bands visualized under UV light (AlphaImager; Quantum Scientific Pty Ltd, Murarrie, QLD, Australia). The *MyoD*-Cre × Z/AP mice were genotyped with primers for *Cre *(5'-CATCGTCGGTCCGGGCTGCC-3' and 3'-CCCCCATGGCTAAGTGCCTTC-5') and *hAP *(5'-ATCGCTGATTTGTGTAGTCGGT-3' and 3'-CAACAGTTGCGCAGCCTGAATG-5') using standard PCR conditions. The genotyping reaction is based on the Taq DNA polymerase methodology and was prepared according to the protocol provided by the manufacturer (Roche Diagnostics, Indianapolis, IN, United States).

### Surgical procedures

#### Tibial fracture

Midshaft tibial fractures were created manually by three-point bending with surgical stable removers, as previously reported [14]. Anesthesia was induced by the intraperitoneal injection of ketamine (35 mg/kg) and xylazine (4.5 mg/kg). A small incision was made slightly below the knee and a 30 G needle was inserted. After the fracture was made, its position was confirmed using a digital x-ray machine (Faxitron X-ray Corp., Wheeling, IL, United States). The 30 G needle was then replaced with a 0.3 mm-diameter stainless steel insect pin to provide additional stability with bone end alignment preserved by an experienced surgeon and confirmed by x-ray. For closed fractures, no additional procedures were performed. In open fractures, the fracture site was subsequently opened and the local periosteum stripped for ~3 mm each side of the fracture site. To mimic the traumatic soft tissue injury that is often associated with open fractures, the muscles surrounding the tibial midshaft was repeatedly manually fenestrated using magnum 15 tattoo needles. Wounds were closed using suture material and Vetbond as required. Analgesia was again given by s.c. buprenorphine and mice were monitored daily for the first 3 days. Fracture repair was monitored by weekly radiography (Faxitron x-ray) and in the event where internal fixation had failed (due to pin slippage, bending or breakage) the affected mouse was culled and excluded from subsequent analysis. Mice were euthanized for 1, 2 and 3 week time points.

#### Tibial drill hole

A medial skin incision was made over the proximal metaphysis of the tibia and the proximal medial tibial bone surface was exposed by elevating the covering muscle flap and thorough periosteal stripping. A defect of 1 mm diameter and 1 mm depth was created in the proximal metaphysis of the right tibiae using a spherical 1 mm burr and thoroughly rinsed with sterile saline. After the defect was made, its position was confirmed by digital x-ray. Subsequently, the muscle flap was excised and the skin closed directly over the defect leaving the defect without any muscle coverage. Anesthesia and analgesia were given as described for the tibial fracture model. Mice were euthanized for hind limb harvest at day 7 after surgery for histochemical staining.

### Histochemical and immunohistochemical staining

#### Human alkaline phosphatase (hAP) staining

Specimens were harvested and processed for cryo-preservation. Samples were initially fixed in 0.25% glutaraldehyde at 4°C overnight and then decalcified in 0.4 M EDTA for 2-3 weeks at 4°C. Samples were then prepared for cryo-preservation by overnight incubation in a 30% sucrose solution, embedded in TissueTek O.C.T. compound (ProSciTech, Kirwan, QLD, Australia) and frozen in liquid nitrogen-cooled isopentane. 5 μm sections were cut on the Leica cryostat and collected on Superfrost slides (HD Scientific). For staining, samples were fixed with 4% paraformaldehyde (PFA) in phosphate buffered saline (PBS) for 20 min at room temperature. Slides were washed in PBS and then heat inactivated at 75°C to eliminate endogenous alkaline phosphatase staining. A NBT/BCIP solution (Roche Diagnostics, Cat # 1161451001) was diluted 1:50 in NBT/BCIP buffer (0.1 M NaCl, 0.1 M Tris-HCl pH 9.4, 0.1 M MgCl_2_, 0.1% Tween-20) and were used to stain hAP^+ ^cells blue, and sections were briefly counterstained with Nuclear Fast Red. Stained slides were taken through an alcohol/xylene series and mounted. Z/AP littermates were included in all staining protocols to produce controls that would stain negative for hAP.

#### Collagen I and II immunohistochemistry

Collagen I and II antibodies were used to detect mature bone cells and chondrocytes respectively using standard protocols. Briefly, cryosections were fixed with 4% PFA and endogenous peroxides were blocked using 0.3% H_2_O_2 _in methanol at room temperature for 10 min. Nonspecific binding of immunoglobulin was blocked by incubating slides in 2% BSA for 20 min. Sections were incubated with the primary antibodies (Collagen I, 1:200; Collagen II, 1:200) for 2 hours at room temperature. Sections were incubated with the anti-rabbit secondary antibody (1:100). All antibodies were purchased from Abcam. Antibody complexes were detected using diaminobenzidine (DAB) substrate (Vector Laboratories, Burlingame, CA, United States). All sections were counterstained with hematoxylin for 15 sec, cleared in ethanol and xylene and mounted.

### Quantification and statistical analyses

**Figure 1:**
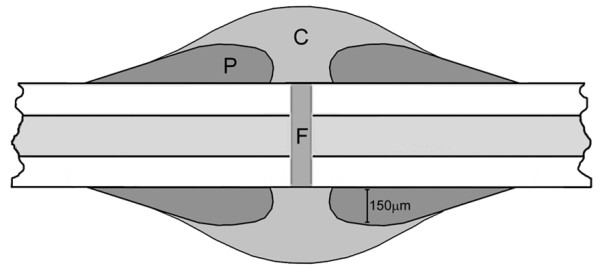
**Schematic representation of the fracture areas used for quantification**. For cell counting analyses, regions of the fracture callus were designated as shown. The fracture gap (F) was defined as the area between the bone ends where the initial impact that broke the endogenous bone occurred. The peri-cortical bone (P) was defined as the area 150 μm above the endogenous bone and stretched along the length of the endogenous bone in the four quadrants shown. The callus area (C) was defined as the rest of the newly formed bone that enveloped the endogenous bone at the breakage point.

Stained tissue sections were scanned using Scanscope virtual scanning software (Aperio). Five grids (350 μm × 350 μm^2 ^in area) were randomly placed around a section of fracture callus. The number of positive cells were counted and expressed as a percentage over the total number of cells in the grid. Cells are expressed as percent averages of five sections from three different animals at the chondrogenic (2 week) and osteogenic (3 week) stages of fracture repair. To quantify the contribution of hAP^+ ^MyoD-lineage cells to fracture repair, fractured bones were divided into separate regions shown schematically in Figure 1. The gap between the two bone ends where the bone was surgically broken was defined as the fracture gap (F), while the granulation tissue that bridged the fracture site was labeled the callus area (C). The peri-cortical bone (P) was identified as the osteoid formed along the bone surface within 150 μm of the tibiae. This region was designated as peri-cortical, as opposed to periosteal, to reflect the surgical stripping of the cellular periosteum normally present on the bone surface in the open fracture group. All cells within the fracture gap and peri-cortical bone were counted. Due to the large size of the callus area, grids (350 μm × 350 μm^2 ^in area) were randomly placed around the callus region and the number of positive cells was expressed as a percentage over the total number of cells in the grid. No cells were counted during the inflammatory/mesenchymal (1 week) phase of fracture repair.

Due to group numbers being less than 10, stringent non-parametric statistical tests were employed. The differences between multiple treatment groups at each time point were analyzed using a Kruskal-Wallis test, and specific groups were compared with a Mann-Whitney U test with values of P ≤ 0.05 considered statistically significant. Statistical tests were performed using SPSS Statistics version 17 (SPSS Inc., Chicago, IL, United States).

## Results

### MyoD-lineage cellular contribution to muscle and non-muscle tissues

Before performing any bone repair experiments, we performed a detailed study to examine any non-specific or developmental expression of the *MyoD*-Cre transgene. Developmental expression was examined in E13.5, E15.0 and E19.0 *MyoD*-Cre^+^:Z/AP^+ ^mouse embryos. In embryo sections, hAP reporter staining was restricted to the mononuclear precursors surrounding the developing skeleton (E13.5) and later as multinucleated muscle fibers (E15.0, E19.0) (Additional File 1). The developmental profile of the hAP reporter indicated a high specificity for muscle.

A range of tissues from skeletally mature mice were then stained to examine any leakage of the promoter or any significant contribution of MyoD-lineage cells to non-muscle tissues. No hAP reporter positive cells were observed in any of these tissues, including bone, bone marrow (Additional File 2), and periosteum (Additional File 3). These data indicate, under normal developmental and physiological conditions, that expression of the *MyoD*-Cre transgene is restricted to the developing and mature muscle. Furthermore, muscle fibers were ubiquitously stained, confirming the role of MyoD as a master regulator of muscle and indicating that a majority of myogenic cells underwent Cre-mediated recombination.

### MyoD-lineage cells contribute to open but not closed fracture repair

To examine the contribution of MyoD-lineage cells in models of bone repair, both *MyoD*-Cre^+^:Z/AP^+ ^mice and *MyoD*-Cre^-^:Z/AP^+ ^(Z/AP^+^) staining control littermates underwent fracture surgery. Both the closed and the open tibial fracture models were reproducible and all animals survived the surgery. Fractures were examined over a time course of 3 weeks and X-rays illustrated that all fractures were bridged by week 3. Fracture repair is rapid in the mouse and studies were not powered to examine differences in healing rates between closed and open tibial fractures. Mice with excessive rotation (defined as 45° or more) and comminuted fractures were excluded from subsequent analysis.

**Figure 2:**
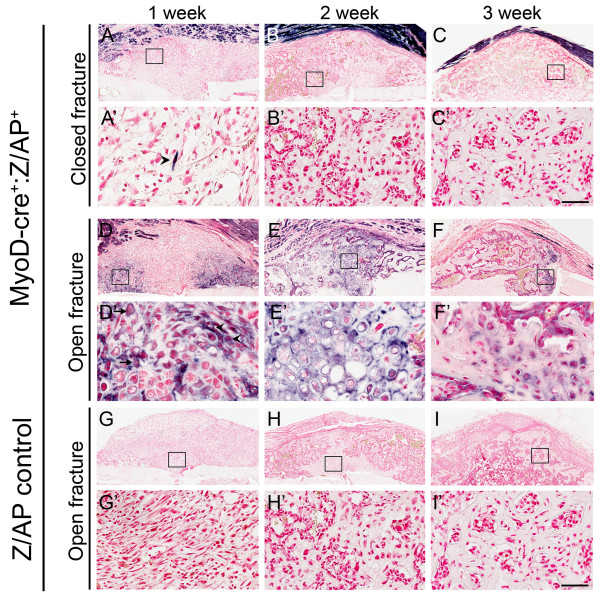
**MyoD-lineage cells contribute to open but not closed fracture repair**. Histologically, only the occasional hAP*^+ ^*labeled cell was detected in the early repair matrix in the closed fracture model **(arrowhead in A')**. No contribution from *MyoD *cells were seen in the latter stages of closed fracture repair **(B-C)**. In the open fracture model, hAP^+ ^cells resembling mesenchymal cells (arrowheads) and chondroblasts (arrows) were observed in the 1 week fracture template **(D, enlarged in D')**. Numerous hAP*^+ ^*cells were visible in the fracture callus at 2 weeks **(E) **and although reporter expression decreased by 3 weeks, hAP*^+ ^*labeled cells were still observed **(F)**. No staining was observed in endogenous bone, or in the fracture calluses of Z/AP control mice **(G-I)**. Scale bar = 100 μm.

The closed fracture model produced minimal damage to the surrounding tissues and featured a largely intact periosteum. Stained sections showed no or minimal number of hAP^+ ^cells throughout the 3 week repair period. At week 1, few hAP^+ ^cells were seen in the mesenchymal template (Figure 2A, arrowhead in 2A'). From 2 weeks onwards, no labeled cells were observed (Figure 2B-C). These results indicate that MyoD-lineage cells make a minimal contribution to closed fracture healing.

In the open fracture model, the fracture site was opened surgically and the periosteum stripped from the cortical bone. The muscle surrounding the tibia was fenestrated to induce substantive soft tissue trauma. In contrast to the closed fracture model, a major contribution by MyoD-lineage cells was observed at all stages of the fracture repair process. At week 1, numerous hAP^+ ^cells were noted in the initial mesenchymal template, particularly in the peri-cortical region (Figure 2D, enlarged in 2D'). This included cells with an elongated mesenchymal morphology (arrow heads) and rounded cells reminiscent of chondroblasts (small arrows). By 2 weeks, a soft fracture callus had formed and cells had the distinctive rounded appearance of chondrocytes. A large proportion of these cells were hAP^+ ^both at the fractured site and throughout the fracture callus (Figure 2E). At 3 weeks, hAP^+ ^osteoblasts were seen on the bone surface and hAP^+ ^osteocytes were observed embedded in the immature woven bone. The reporter positive cells at week 3 were mainly present in the callus interior, and the exterior remained mainly reporter negative (Figure 2F).

No hAP staining was observed in any of the controls samples taken from mice possessing only the Z/AP transgene (Figure 2G-I). This indicates that heat inactivation of endogenous alkaline phosphatase was complete and that all enzymatic staining was specific to the *MyoD-*Cre and Z/AP transgenes. As an additional internal control, no hAP reporter staining was observed in any of the non-fractured bone of *MyoD-*Cre^+^:Z/AP^+ ^mice in either the closed or open fracture models.

To quantify the contribution of MyoD-lineage cells to open and closed fracture healing, whole callus sections were digitally imaged using a Scanscope slide scanner. Regional quantification was performed in week 2 and week 3 healing fractures (Table 1). For open fractures, the greatest contribution was seen in areas of early bone repair such as in the fracture gap where up to 53% of cells were hAP^+^. Reporter cell numbers were found to decrease in areas away from the initial fracture and also decrease over time.

**Table 1:** MyoD-lineage contribution to open fracture repair (% of hAP+ cells)

	Fracture location		
	**Fracture gap (F)**	**Peri-cortical bone (P)**	**Callus area (C)**

**2 week**	53.9 ± 4.7	40.0 ± 2.0	35.9 ± 4.5

**3 week**	47.0 ± 4.7	21.3 ± 2.0	13.2 ± 1.3

**Figure 3:**
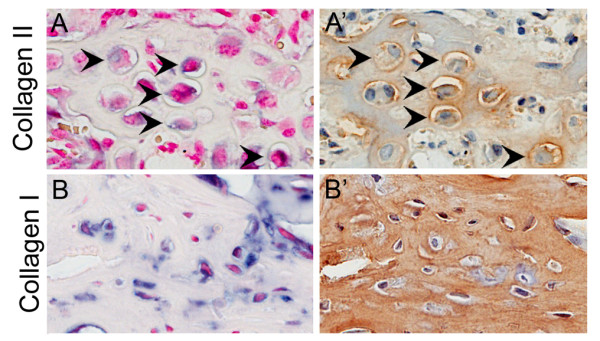
**hAP*^+ ^*cells express chondrogenic and osteogenic markers**. Serial sections were stained for hAP^+ ^cells **(A, B) **and type II collagen in fractures at week 2 **(A') **or type I collagen in the peri-cortical region of open fracture samples at week 3 **(B')**. Collagen staining was seen in both the cells and the matrix. hAP^+ ^cells were found to stain with collagen markers (**arrowheads**) indicating that formally myogenic progenitors had contributed to mature cartilage/bone tissue. Scale bar = 50 μm.

To examine whether the MyoD-lineage cells were having a productive role in the bone healing process, hAP^+ ^cells found in areas of bone and cartilage were co-stained for bone/cartilage markers (type I or type II collagen) on serial sections. Immunohistochemical staining confirmed that hAP^+ ^labeled cells had progressed to express characteristic cartilage and bone extracellular matrix proteins (Figure 3).

### Endogenous osteoprogenitors do not express MyoD during bone defect repair

**Figure 4:**
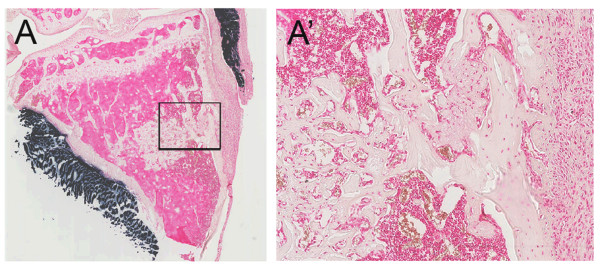
**Endogenous osteoprogenitors do not express MyoD during tibial defect repair**. Decalcified sections from drill hole defects (day 7 of repair) were stained for hAP+ cells. No MyoD-lineage cells were present within the defect (**A, A'**) but were present within the tibialis anterior muscle (**A**).

The aforementioned experiments suggested that MyoD-lineage cells from the muscle migrated and repaired open fractures in the absence of other osteocompetent cells. An alternative scenario was that cells within open fractures originating from the bone spontaneously expressed MyoD during osteogenic differentiation. To exclude this possibility, we performed a drill hole defect where local muscle was physically stripped and endogenous osteoprogenitors were plentiful. In this system no staining was observed within the healing defects (Figure 4).

## Discussion

These orthopaedic bone repair studies offer new and valuable perspectives on the capacity of MyoD-lineage cells to contribute to bone formation and fracture repair. While the conditions associated with recalcitrant fracture repair are well described on a physiological level, the specific cell types that normally contribute to these processes remain poorly defined. Many lineage tracing studies described in the literature are complicated by issues of transient reporter expression. In the current study, the Cre/*loxP *system was adopted to permanently label MyoD-lineage cells and effectively track their contribution to bone. Initial studies were performed using the ROSA26R reporter strain (data not shown), however false positive staining in osteoclasts emerged as a significantly confounding factor [15]. The Z/AP reporter strain proved to be a superior system with negligible background in adult mouse bone. Expression of hAP in MyoD-lineage cells did not appear to impair or augment fracture healing based on union or callus size by x-ray.

The *MyoD*-Cre reporter mouse line was selected due to its apparent specificity to myogenic cells. *MyoD *is a specific marker of myogenic cells and is expressed in the embryonic myotome from as early as embryonic day 9.5 (E9.5) [16]. In muscle satellite cells, *MyoD *expression is initiated after satellite cell activation [17-19], however recent work by Kanisicak et al., demonstrated that all satellite cell progenitors pass through a *MyoD*-expressing stage sometime during their developmental history [20]. While Goldhamer *et al *reported rare staining in skeletal tissues including the perichondrium and several osteocytes, it is unclear whether this was due to transgene "leakiness" or due to a rare early developmental transdifferentiation event involving the MyoD-lineage [12]. Nevertheless, our staining in embryonic and adult tissues found transgene expression to be highly restricted to the muscle (Additional Files 1 and 2) and not in the periosteum of adult mice (Additional File 3).

Muscle flaps are often used in orthopaedic surgery and the beneficial effects of muscle proximity to fracture healing are often attributed to the high vascularity of this tissue. In contrast, we have hypothesized that in fracture repair muscle may make a direct cellular contribution to bone, acting as a "secondary periosteum" in instances where the periosteum itself is damaged or absent [4]. To test this, we utilized two fracture models: a closed fracture model where the periosteum was largely intact and an open fracture where the bone was denuded of the periosteum with the adjacent muscle damaged.

Consistent with our hypothesis, MyoD-lineage cells made substantial contributions to every stage of the open fracture repair cascade. The greatest number of hAP^+ ^cells in the fractured bones were located in areas closest to the initial site of impact (i.e., at the fracture gap > peri-cortical bone > external callus area) (Table 1). This could suggest that MyoD-lineage cells are one of the first cell types to migrate to the site of injury due to their close proximity to the fractured bone. The concomitant muscle trauma may facilitate the passage of MyoD-lineage cells through injured fascial compartments to the fracture site. The proportion of reporter positive cells in open fractures were found to decrease with time, suggesting that myogenic progenitors can act in the initial bone healing response, but are less involved with subsequent bone remodeling. A model by Harry *et al. *has demonstrated functional benefits for access to the adjacent muscle during mouse tibial fracture healing. When muscle access was physically excluded using a polymer sheath, this strongly reduced fracture healing despite adequate or increased vascularity over exclusion of fasciocutaneous tissue [21,22].

In contrast to the traumatic open fracture scenario, MyoD-lineage cells made an insignificant contribution to closed fracture repair. This may suggest that an intact periosteum can act as a physical barrier to prevent the invasion of myogenic progenitors, and also strengthens the concept that periosteal cells contributing to repair do not transiently express MyoD. The periosteum is a highly osteogenic tissue and, when present, its contribution to bone healing is sufficient and does not require alternative osteoprogenitor sources. The lack of hAP^+ ^cells in closed fractures also argues against the possibility of spontaneous expression of the *MyoD-*Cre transgene by nascent osteoprogenitor populations responsible for repair (including periosteal cells, Additional File 3). To further examine whether MyoD-lineage cells could migrate into and contribute to bone healing, we tested a tibial defect model where the adjacent musculature had been stripped. No hAP+ staining cells were observed in the healing defect supporting the hypothesis that muscle accessibility is important for the contribution of MyoD-lineage cells.

## Conclusion

In summary, these studies show for the first time that a population or sub-population of myogenic progenitors of the MyoD-lineage can make a significant cellular contribution to bone repair. In a fracture repair setting, this required disruption of the periosteum and trauma to the local tissues. In a defect repair setting, this required direct access to the muscle. These data suggest that the recruitment of MyoD-lineage cells can be highly dependent on the nature and location of the bone injury. Even in the surgical mouse models with the greatest myogenic progenitor contribution, approximately half of the cells originated from non-myogenic lineages. Thus cell lineages other than myogenic cells including vascular cells are also likely to have a major role in bone formation and repair [23].

Apart from scientific clarification of the cellular contribution to bone repair in other orthopaedic models, future studies can aim to manipulate the surgical systems with the aim of maximizing MyoD-lineage cell access and mobilization to augment repair. Methods which optimize the contribution of secondary (non-periosteal) osteoprogenitors may be translatable to clinical practice and play a future role in improving the union rates of high energy and open fractures.

## Competing interests

The authors declare that they have no competing interests.

## Authors' contributions

RL was the primary researcher involved with study conception and fracture surgery, characterization of the mouse model, and data analysis. OB was responsible for the tibial defect model. LP and KM performed peri-operative anaesthesia and post-surgical monitoring. AM provided assistance with histological analyses. AS and DL oversaw the project, and with RL, were responsible for drafting the manuscript, interpretation of data, and manuscript writing. All authors read and approved the final manuscript.

## Pre-publication history

The pre-publication history for this paper can be accessed here:

http://www.biomedcentral.com/1471-2474/12/288/prepub

## Supplementary Material

Additional file 1 **MyoD-lineage cells are developmentally restricted to the musculature**. Strong hAP staining was observed in mononuclear cells surrounding the developing skeleton at E13.5 (**A**). As development progressed, these cells fused into multinucleated myofibers surrounding the ribs (**B**) and the developing limb buds (**C**). No hAP^+ ^cells were observed in any skeletal elements throughout the developmental time frames studied. hAP expression was limited to the muscles of skeletally mature mice (**D**) and no staining was observed in any bony elements (**E**). Scale bar = 100 μm. Click here for file

Additional File 2 **MyoD-lineage cells do not contribute to adult non-muscle tissues**. No hAP staining was found in non-muscle tissues including kidney (**A**), liver (**B**), lung (**C**), spleen (**D**), and heart (**E**). Strong and universal staining was observed in all skeletal muscle fibers (**F**). Click here for file

Additional File 3 **MyoD-lineage cells are not found in the periosteum**. No hAP staining was seen in the periosteum (arrowheads), underlying bone (B) or bone marrow (BM). The adjacent muscle (M) stained positive. Click here for file
