# Supplementary figures and images for: Myogenic progenitors contribute to open but not closed fracture repair
Source: BMC Musculoskelet Disord. 2011 Dec 22;12:288. doi: 10.1186/1471-2474-12-288 (PMC3266223; doi:10.1186/1471-2474-12-288)

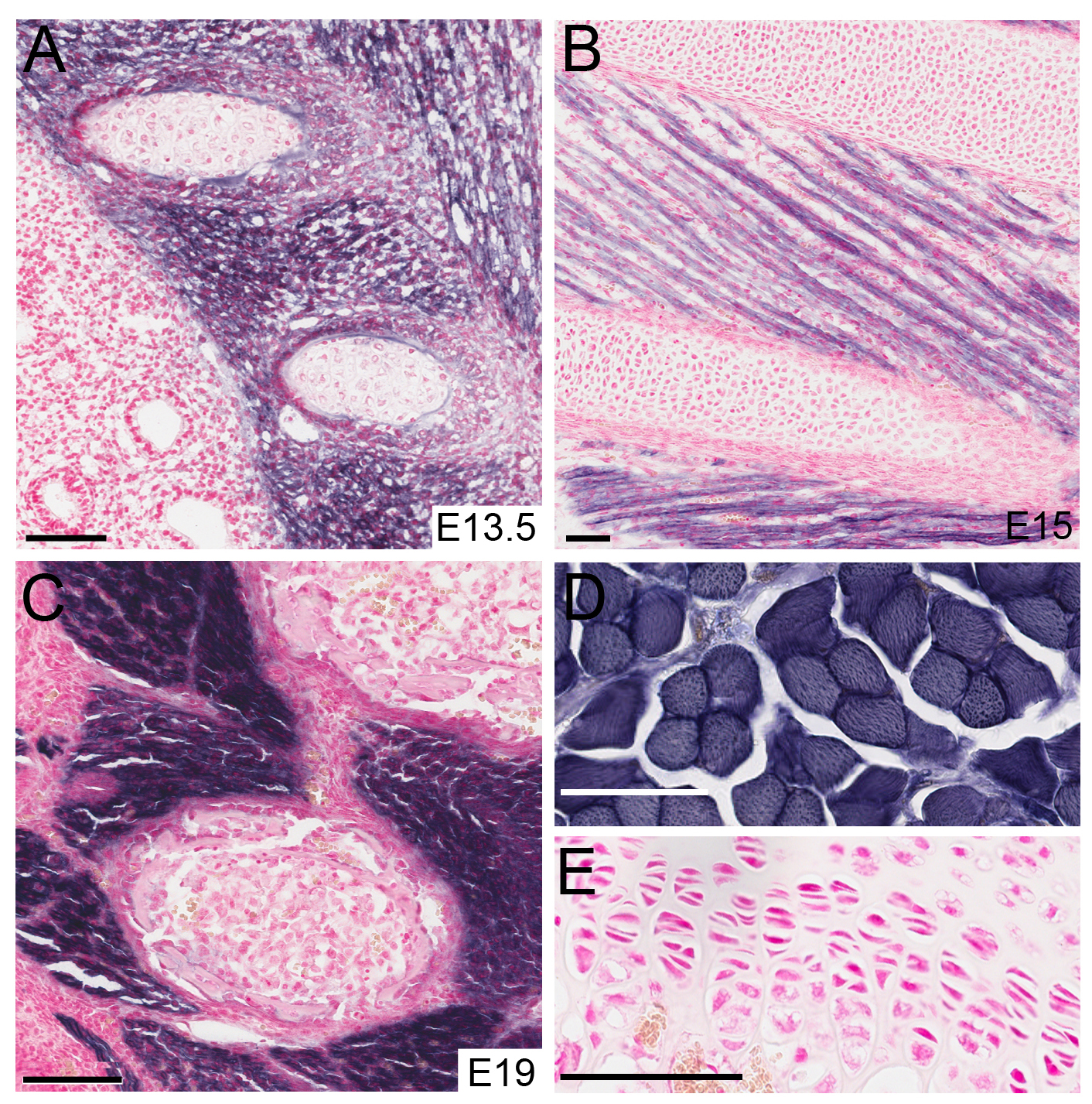

Supplement: Additional file 1 — MyoD-lineage cells are developmentally restricted to the musculature. Strong hAP staining was observed in mononuclear cells surrounding the developing skeleton at E13.5 (A). As development progressed, these cells fused into multinucleated myofibers surrounding the ribs (B) and the developing limb buds (C). No hAP+ cells were observed in any skeletal elements throughout the developmental time frames studied. hAP expression was limited to the muscles of skeletally mature mice (D) and no staining was observed in any bony elements (E). Scale bar = 100 μm. [file 1471-2474-12-288-S1.JPEG]

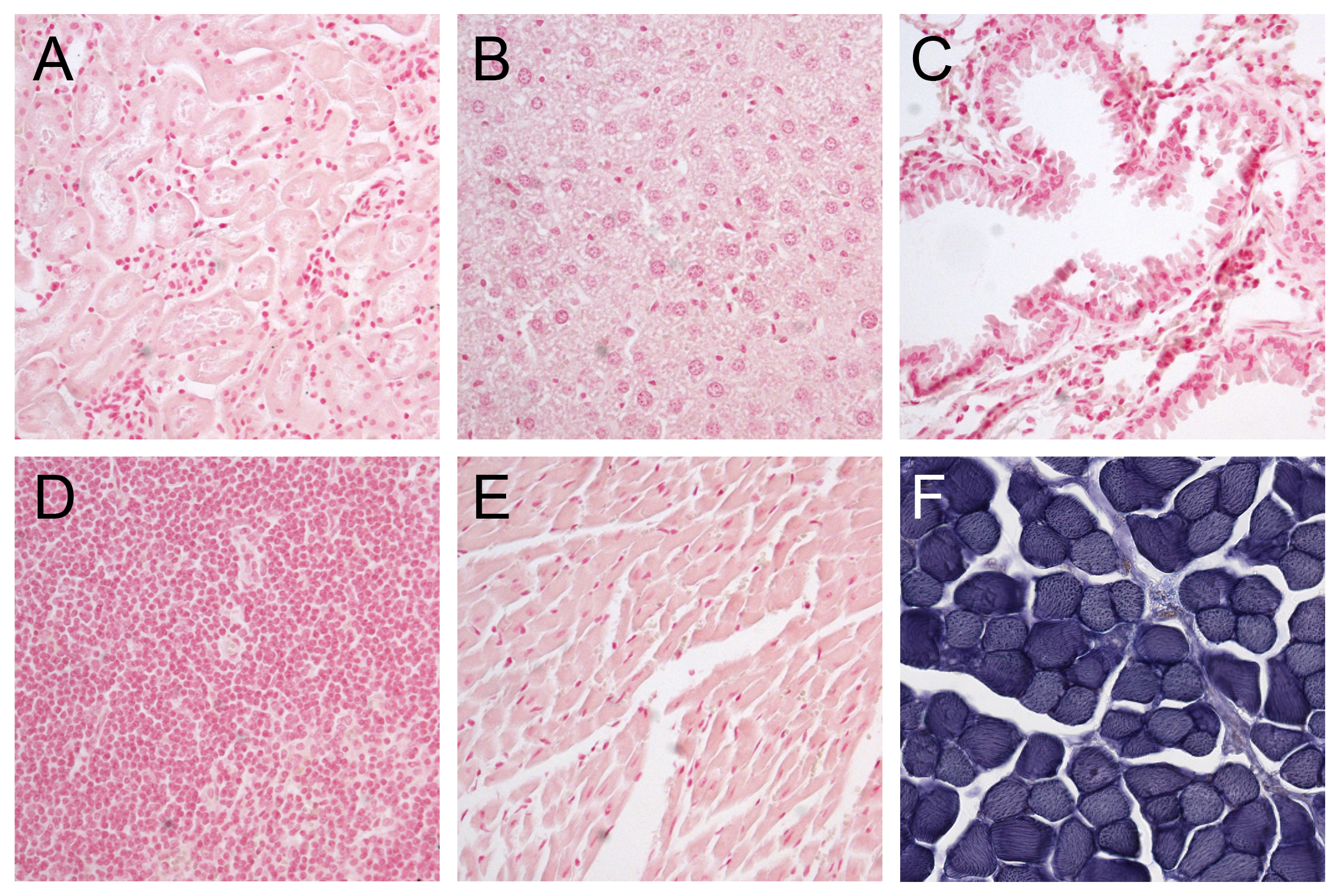

Supplement: Additional File 2 — MyoD-lineage cells do not contribute to adult non-muscle tissues. No hAP staining was found in non-muscle tissues including kidney (A), liver (B), lung (C), spleen (D), and heart (E). Strong and universal staining was observed in all skeletal muscle fibers (F). [file 1471-2474-12-288-S2.JPEG]

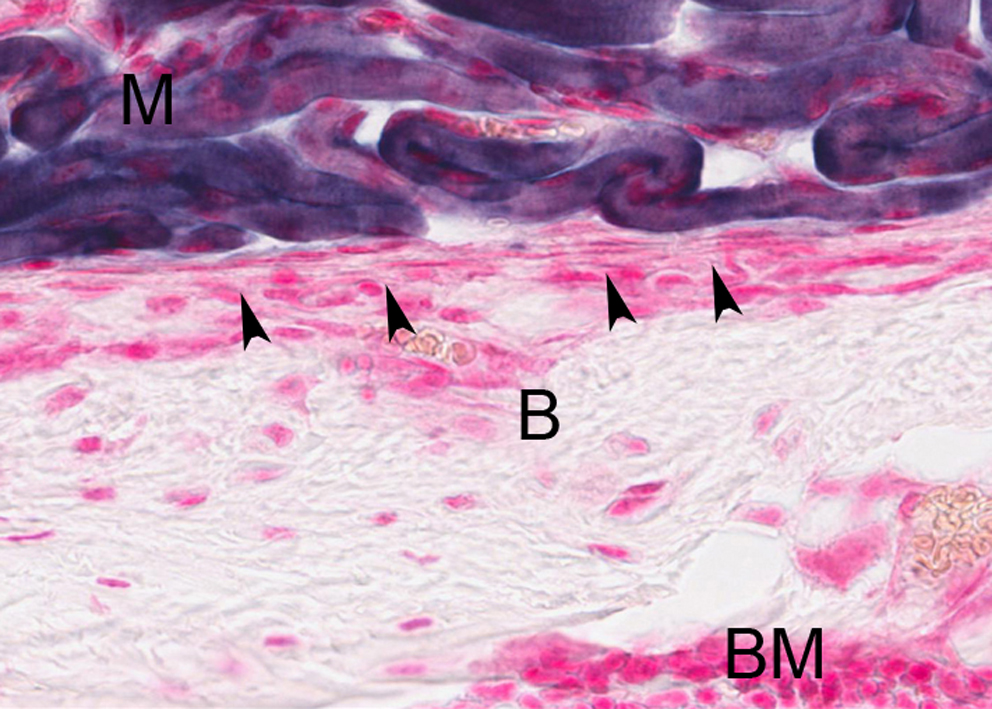

Supplement: Additional File 3 — MyoD-lineage cells are not found in the periosteum. No hAP staining was seen in the periosteum (arrowheads), underlying bone (B) or bone marrow (BM). The adjacent muscle (M) stained positive. [file 1471-2474-12-288-S3.JPEG]
